# Evaluation of Antigen-Specific IgM and IgG Production during an *In Vitro* Peripheral Blood Mononuclear Cell Culture Assay

**DOI:** 10.3389/fimmu.2017.00794

**Published:** 2017-07-10

**Authors:** Yoshiko Matsuda, Ryoichi Imamura, Shiro Takahara

**Affiliations:** ^1^Department of Advanced Technology for Transplantation, Graduate School of Medicine, Osaka University, Osaka, Japan; ^2^Department of Urology, Graduate School of Medicine, Osaka University, Osaka, Japan

**Keywords:** *in vitro* assay, antibody-associated disease, IgM memory B cells, IgG memory B cells, germinal centers

## Abstract

The recent attention given to diseases associated with memory B-cell (mBC)-produced antibodies (Abs) suggests the need for a similar *in vitro* assay to evaluate the functions of mBCs. Here, we cultured peripheral blood mononuclear cells (PBMCs) with the intent to collect mBC-derived Abs *in vitro* and maintain their cell–cell contact-dependent interactions with helper T-cells. PBMCs were cultured with interleukin (IL)-21, CpG-oligodeoxynucleotides (ODN), phorbol myristate acetate (PMA), and phytohemagglutinin/leucoagglutinin (PHA-L) in 24-well flat-bottom plates (5 × 10^5^ cells/well). A culture supernatant analysis of PBMCs from healthy donors (*n* = 10) indicated that antigen-specific IgM Ab levels in a PBMC culture supernatant might be better able to demonstrate the antigen sensitization status in a smaller peripheral blood sample, compared to IgG because Epstein–Barr virus-specific IgM mBCs circulate peripherally at a significantly higher frequency once antiviral humoral immunity has stabilized. Thus, our *in vitro* assay demonstrated the potential significance of antigen-specific IgM Ab production in the culture supernatants. Furthermore, an analysis of cultured PBMCs from allograft kidney recipients (*n* = 16) sensitized with *de novo* donor-specific human leukocyte antigen (HLA)-specific Abs (DSAs) showed that IgM-type HLA-specific Abs were detected mainly from the culture supernatants from PBMCs of patients with stable graft function, whereas IgG isotype HLA Abs were detectable only from patients with biopsy-proven antibody-mediated rejection. In other words, these IgG isotype Abs also represented an activated humoral immune response *in vivo*. Additionally, IgM- and IgG-expressing mBCs from healthy donors (*n* = 5) were cultured with IL-21, CpG-ODN, and a supernatant produced by stimulating CD19^+^ B-cell-depleted PBMCs with PHA-L and PMA in 24-well flat-bottom plates (1 × 10^5^ cells/well), and the resulting *in vitro* analysis provided some information regarding the biological processes of IgG and IgM mBCs in peripheral blood. Taken together, our findings suggest that antigen-specific Ab subtype analyses of supernatants from cultured PBMCs might more effectively and accurately reflect a patient’s Ab-associated pathological condition vs. than serum IgG and IgM levels.

## Introduction

Antigen-specific antibodies (Abs) are produced by memory B-cell (mBC)-derived plasma cells (PCs). Furthermore, some reports indicate that no available immunosuppressive agent can control PCs growth and survival. Therefore, an understanding of Ab-associated disease first requires an understanding of the biological processes that underlie the growth and survival of mBCs.

Briefly, B-cells initially develop in the bone marrow. Here, highly self-reactive immature B-cells are deleted, and the remaining cells exit the bone marrow to the peripheral circulation. During the transitional stage of B-cell differentiation, cells that express self-antigen-reactive B-cell receptors (BCRs) are subjected to clone deletion, BCR editing, anergy, and immunological ignorance (1).

The stimulation of BCRs on naïve B-cells in the peripheral lymphoid tissue *via* receptor cross-linking induces clonal B-cell expansion and antigen uptake. Subsequently, this antigen is presented in combination with a major histocompatibility complex class II molecule on the naïve B-cell surface for recognition by helper T-cells. Subsequently, activated naïve B-cells and accompanying T-cells migrate into primary lymphoid follicles and subsequently form germinal centers (GCs) in secondary lymphoid tissues (2).

Within GCs, activated naïve B-cells undergo somatic hypermutation (SHM) of the variable regions and class-switch recombination (CSR) of immunoglobulin-encoding genes and differentiate into mBCs or PCs. During this process, mBCs with higher affinities for non-self-antigens are selected and mBCs with low affinities are deleted. The remaining mBCs differentiate into PCs (3–6).

Previous research regarding Ab-associated diseases has mainly focused on antigen-specific IgGs as the etiologic agent. IgG-producing mBCs differentiate in GCs after undergoing SHM and CSR. These cells are localized in lymph nodes near the primary infection site and can more rapidly differentiate into PCs, compared with IgM-producing mBCs (7, 8). Furthermore, these mBCs-derived IgGs cause tissue injury by absorption into target antigen in context of Ab-associated diseases.

By contrast, the clinical significance of antigen-specific IgM with respect to Ab-associated diseases remains controversial.

Many reports have indicated that IgM mBCs can be subclassified as having either the IgD^−^ or IgD^+^ phenotype. IgM (IgD^−^) mBCs, which do not develop in GCs (9), respond in an extra-follicular, thymus-independent manner and produce natural Abs with lower affinities for antigens (10). By contrast, IgM (IgD^+^) mBCs undergo SHM in GCs and differentiate into PCs that produce sufficient amounts of Abs specific for thymus-dependent antigens. These latter somatically mutated IgM mBCs have been reported to exhibit similar functional capacities to those of IgG mBCs (11).

Various types of Ab isotypes have elicited research interest. IgG-type DSAs have received considerable attention in the field of organ transplantation. Regarding autoimmune diseases, serum levels of self-antigen-specific IgM and IgG have been used to evaluate pathological conditions (12, 13). In the field of viral infection, both IgG and IgM viral antigen-specific Abs have been used to evaluate previous or current infection status, and IgM production has been recognized as an early diagnostic parameter (14, 15).

Accordingly, the role of mBC-derived antigen-specific IgM Abs in Ab-associated diseases should be elucidated further using *in vitro* assays of supernatants, similar to those used to study T-cells. In our study, we attempted to develop an *in vitro* assay method enabling us to collect mBC-derived Abs to possibly elucidate the biological processes of antigen-specific IgG and IgM mBCs in peripheral blood. We further aimed to establish a culture supernatant analysis to provide some information about the potential of each type of Ab associated with a pathological condition in context with Ab-associated diseases.

## Materials and Methods

### Participants and Samples

This study followed the principles of the Declaration of Helsinki, and all subjects provided informed consent to participate. Peripheral blood (8 ml) samples were collected from 10 healthy donors (3 men and 7 women; average age, 41.4 ± 8.8 years; range: 31–55 years) and 16 kidney-allograft recipients sensitized *de novo* to donor-specific antigens (8 men and 8 women; average age, 46.3 ± 17.0 years; age range: 17–77 years). Additionally, CD27^+^ mBCs and CD27^−^ naïve B-cells, as well as IgM mBCs and IgG mBCs, were separated from the peripheral blood mononuclear cells (PBMCs) of the five healthy donors (four men and one woman; average age, 39.3 ± 9.7 years; age range: 24–49 years).

### Isolation and Culture of PBMCs

Peripheral blood mononuclear cells were isolated over a Ficoll-Hypaque density gradient (Sigma-Aldrich, St. Louis, MO, USA) and cultured in 24-well flat-bottomed plates (5 × 10^5^ cells/well) in basal B-cell culture medium [Iscove’s modified Dulbecco’s medium (Sigma-Aldrich) supplemented with 10% fetal calf serum (FCS; Thermo Scientific HyClone, Logan, UT, USA), 50 µg/ml human transferrin–selenium, and 5 µg/ml human insulin (Gibco/Invitrogen Co., Carlsbad, CA, USA)]. Cells were cultured in the presence of the following additives: 50 ng/ml interleukin (IL)-21, 2.5 µg/ml phosphorothioate CpG-oligodeoxynucleotides (ODN) 2006, 2.5 µg/ml phytohemagglutinin/leucoagglutinin (PHA-L), and 15 ng/ml phorbol myristate acetate (PMA). For IgM-BCR cross-linking, we cultured PBMCs (5 × 10^5^ cells/well, 24-well flat-bottomed plate) in medium supplemented with Affini Pure F(ab)^2^ Fragment Goat antihuman IgM (5.2 µg/ml).

### Isolation and Culture of CD27^+^ mBCs and CD27^−^ Naïve B-Cells

To isolate mBCs from naïve B-cells, PBMCs were subjected to negative selection using a B-cell isolation kit (Miltenyi Biotech, Auburn, CA, USA). Unlabeled B-cells were passed through the column, washed with buffer, and subjected to magnetic activated cell sorting using CD27-conjugated microbeads according to the manufacturer’s instructions. The isolated mBCs were cultured in 24-well flat-bottom plates (1 × 10^5^ cells/well) in basal B-cell culture medium supplemented as follows: 50 ng/ml IL-21, 2.5 µg/ml phosphorothioate CpG-ODN 2006, CD40 ligand, and 5 µg/ml anti-polyhistidine monoclonal antibody (mAb). The isolated B-cells were cultured without helper T-cells, which express CD40 ligand or humoral factors produced by these cells. Therefore, it was necessary to supplement the culture with soluble CD40 ligand to support B-cell growth and survival.

### Isolation and Culture of IgG mBCs and IgM mBCs

IgG mBCs and IgM mBCs were further separated using IgG and IgM mBC isolation kits (Miltenyi Biotech) according to the manufacturer’s instructions. The isotype-specific mBCs were cultured in 24-well flat-bottom plates (1 × 10^5^ cells/well) in basal B-cell culture medium supplemented as follows: 50 ng/ml IL-21, 2.5 µg/ml phosphorothioate CpG-ODN 2006, and 5% PBMC supernatant; the latter was produced by stimulating CD19^+^ B-cell-depleted PBMCs obtained from healthy male donors (*n* = 5) for 36 h in the presence of 15 ng/ml PMA and 2.5 µg/ml PHA-L in RPMI-1640 supplemented with 5% FCS. For IgM-BCR cross-linking, we cultured PBMCs (5 × 10^5^ cells/well, 24-well flat-bottomed plate) in basal B-cell culture medium supplemented with Affini Pure F(ab)^2^ Fragment Goat antihuman IgM (5.2 µg/ml).

### Reagents

Recombinant human IL-21 was purchased from Miltenyi Biotech. Histidine-tagged soluble recombinant human CD40 ligand, anti-polyhistidine mAb, and human recombinant a proliferation-inducing ligand (APRIL) were obtained from R&D Systems (Minneapolis, MN, USA). Phosphorothioate CpG-ODN 2006 was purchased from Invivogen (San Diego, CA, USA). PHA-L and PMA were purchased from Sigma-Aldrich (St. Louis, MO, USA). The Affini Pure F (ab)^2^ fragment goat antihuman IgM used for IgM-BCR cross-linking was purchased from Jackson Immunoresearch Laboratories (West Grove, PA, USA). A Mem-PER™ Plus Membrane Protein Extraction Kit from Thermo Scientific (Waltham, MA, USA) was used for membrane extraction.

### Flow Cytometric Analysis

To evaluate differentiation, cells were labeled with fluorescein isothiocyanate (FITC)-tagged anti-CD38 (eBioscience, San Diego, CA, USA), phycoerythrin (PE)-tagged, anti-138 and allophycocyanin-tagged anti-CD19 mAbs (BD Biosciences, San Jose, CA, USA). Dead cells were stained with propidium iodide (Sigma-Aldrich). All flow cytometric analyzes were performed on a FACS Calibur dual-laser flow cytometer, and data were analyzed using Cell Quest acquisition/analysis software (BD Biosciences).

### Enzyme-Linked Immunosorbent Assay (ELISA)

For ELISAs, 96-well plates were coated with a recombinant Epstein–Barr virus (HHV-4) p18, GST tag (ProSpec, East Brunswick, NJ, USA) to generate a standard curve and polyclonal F(ab)^2^ goat antihuman IgG heavy chain Abs (Biosource, Camarillo, CA, USA) or affinipure F(ab′)^2^ goat antihuman IgM Fc5μ fragment-specific Abs (Jackson Immunoresearch Laboratories). Coated plates were incubated at 4°C overnight.

Each plate was washed five times with phosphate-buffered saline (PBS; Sigma-Aldrich) containing 0.1% Tween-20. Next, 100 µl aliquots of supernatant from each culture condition, media alone, and IgG standards (calibration curve) were diluted with PBS in the range of 1:200–1:10,000 (IgM standards, 1:1,600–1:80,000), added to the plates, and incubated overnight at 4°C. The plates were then washed five times with PBS containing 0.1% Tween-20 and incubated with horseradish peroxidase-conjugated antihuman IgG or antihuman IgM (Jackson Immunoresearch Laboratories) for 2 h at room temperature. The plates were again washed five times with PBS containing 0.1% Tween-20. After adding tetramethylbenzidine substrate (Thermo Scientific, Rockford, IL, USA) to each well for color development, the optical density in each well was measured at 630 nm using a PowerScan4 Microplate Reader (DS Pharma Medical Co., Ltd., Osaka, Japan); the calibration curve was subsequently generated and used to determine the total IgG/IgM EBV antibody titers and IgG/IgM levels. To determine the IgG and IgM concentrations in the samples, a Human IgG total Ready-SET-GO! and Human IgM total Ready-SET-GO! were purchased from eBioscience.

### Detection of Human Leukocyte Antigen (HLA) Abs in the Culture Supernatants

We collected additional culture supernatant samples and concentrated these by fivefold prior to an Ab screening evaluation based on Flow PRA 60 kits (One Lambda, Canoga Park, CA, USA). Positive cases were subjected to a further Luminex technology-based analysis involving HLA LAB Screen class 1 and/or class 2 single-antigen beads (One Lambda, Canoga Park, CA, USA).

### Statistical Analysis

All data are presented as median values and ranges unless otherwise indicated. An analysis of variance or paired or unpaired Student’s *t*-test was used to evaluate the significance of differences between variables. Prism 7 software (GraphPad Inc., San Diego, CA, USA) was used for statistical analyzes. A two-tailed *p*-value < 0.05 was considered statistically significant.

## Results

### Compared with CD27^−^Naïve B-Cells, CD27^+^ mBCs Differentiate More Rapidly into PCs

**Figure 1:**
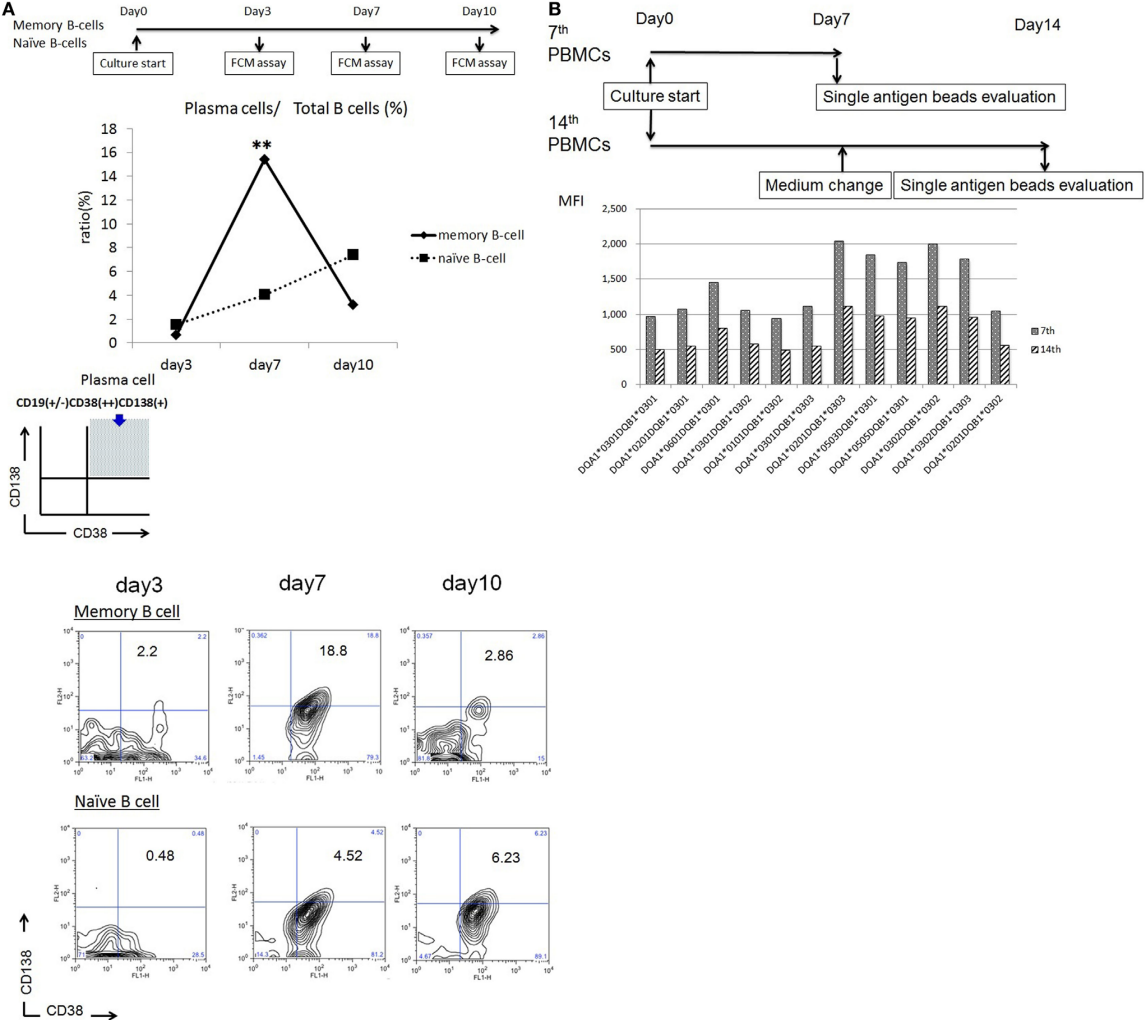
**(A)** Memory (CD27^+^) and naïve (CD27^−^) B cells isolated from single donors were cultured in 24-well flat-bottomed plates (1 × 10^5^ cells/well) in basal B-cell culture medium supplemented as follows: 50 ng/ml interleukin (IL)-21, 2.5 µg/ml phosphorothioate CpG-oligodeoxynucleotide (ODN) 2006, CD40 ligand, and 5 µg/ml anti-polyhistidine monoclonal antibody (mAb) *in vitro*; the cultured cells were stained with fluorescein isothiocyanate -tagged anti-CD38, phycoerythrin -tagged anti-CD138, and allophycocyanin -tagged anti-CD19 mAbs on days 3, 7, and 10; and subjected to flow cytometry analysis. Total B cells were identified as CD19^+^ cells. Plasma cells (PCs) were identified as CD19 ^+/−^CD38^++^CD138^+^ cells. Differentiation of stimulated CD27^+^ memory B-cell into PCs was compared with that of CD27^−^ naïve B cells, as determined by the ratio (%) of PCs to total B cells. Memory (CD27^+^) and naïve (CD27^−^) B cells were collected from five independent healthy donors, and the data represent five independent experiments. **(B)** Peripheral blood mononuclear cells obtained from an allograft recipient sensitized to donor human leukocyte antigen were cultured using 50 ng/ml IL-21 and 2.5 µg/ml phosphorothioate CpG-ODN 2006 in the presence of 2.5 µg/ml phytohemagglutinin/leucoagglutinin and 15 ng/ml phorbol myristate acetate *in vitro* for 1 week, after which the culture medium was replaced under the same conditions, and cells were cultured for another week. Culture supernatants were collected on days 7 and 14 and subjected to evaluation with Luminex single-antigen beads.

*In vitro*-cultured CD27^+^ mBCs and CD27^−^ naïve B-cells were collected after 3, 7, and 10 days and analyzed by flow cytometry analysis. B-cells were identified as CD19^+^ cells. PCs were identified as CD19^+^CD38^++^CD138^+^ cells. Stimulated CD27^+^ mBCs were found to differentiate into PCs significantly earlier compared with CD27^−^ naïve B-cells, as determined by the ratio (%) of PCs/total B-cells on day 7 (15.4 ± 6.2 vs. 4.1 ± 0.6%: *p* < 0.01) (Figure 1A).

### Antigen-Specific mBCs Could Be Stimulated to Differentiate into PCs by Day 7 under Immunosuppression

Peripheral blood mononuclear cells obtained from an allograft recipient sensitized to donor HLA were cultured *in vitro* for 1 week. Following this, the culture medium was replaced and cells were cultured for another week. Culture supernatants were collected on day 14. As a result, HLA Abs produced in the PBMC culture supernatants from days 0 to 7 had a higher concentration than those produced from days 7 to 14 (Figure 1B).

### *In Vitro* Assay Evaluation of IgG/IgM Production in PBMC Culture Supernatant

**Figure 2:**
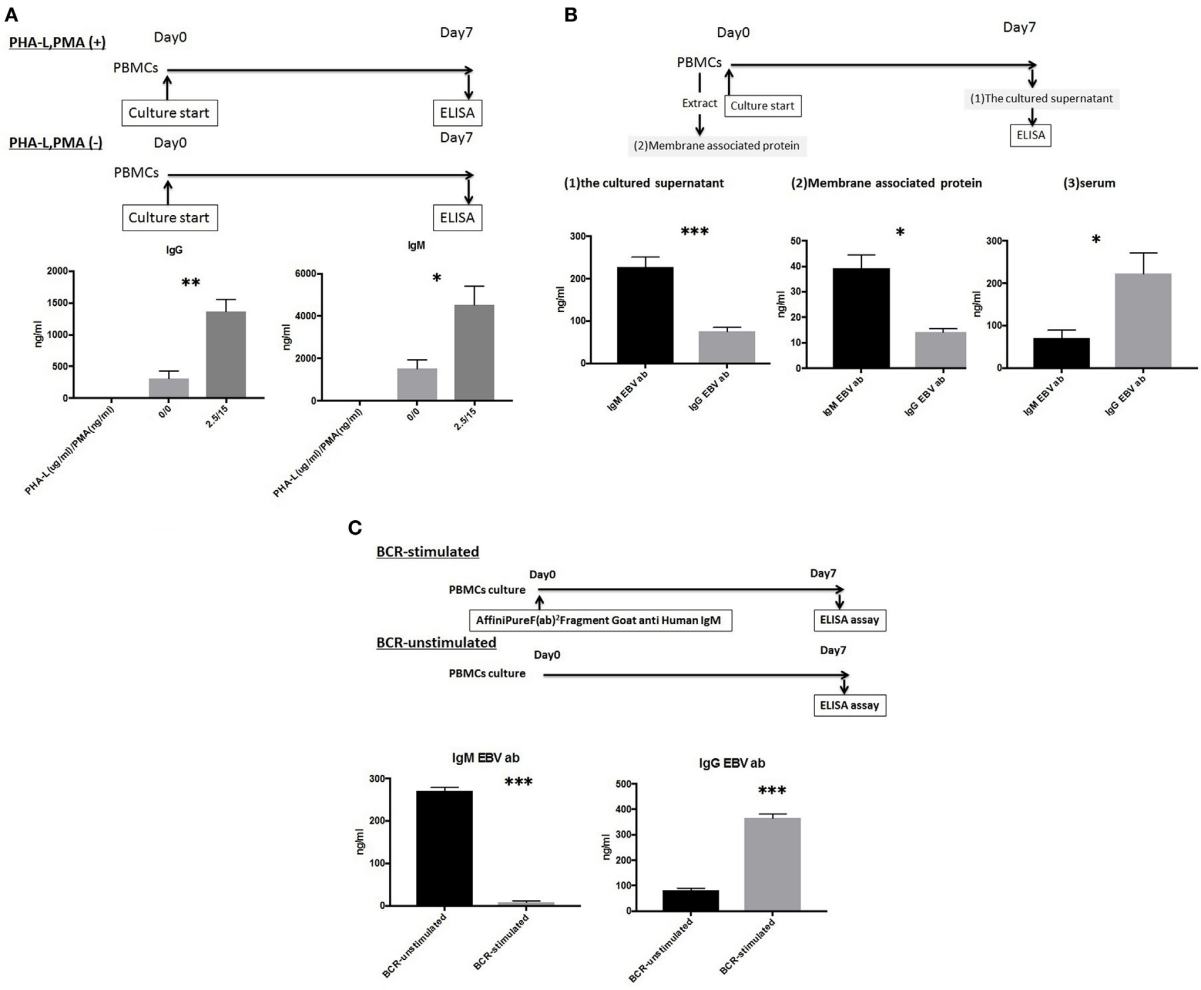
**(A)** Peripheral blood mononuclear cell (PBMCs) from 10 healthy donors were cultured using 50 ng/ml interleukin (IL)-21 and 2.5 µg/ml phosphorothioate CpG-oligodeoxynucleotide (ODN) 2006 and in the presence or absence of 2.5 µg/ml phytohemagglutinin/leucoagglutinin (PHA-L) and 15 ng/ml phorbol myristate acetate (PMA) *in vitro* for 1 week. IgG and IgM levels in the supernatants were quantified using enzyme-linked immunosorbent assay (ELISA). **(B)** PBMCs from 10 healthy donors were cultured using 50 ng/ml IL-21 and 2.5 µg/ml phosphorothioate CpG-ODN 2006 in the presence of 2.5 µg/ml PHA-L and 15 ng/ml PMA *in vitro* for 1 week. EBV-specific IgG and IgM antibodies (Abs) in cell supernatants were quantified using ELISA (1), extracted membrane-associated proteins. Membrane-associated proteins were extracted from non-cultured PBMCs (5 × 10^6^ cells). EBV-specific IgG and IgM Abs in extracted membrane-associated proteins were quantified using ELISA (2), and sera. EBV-specific IgG and IgM Abs in serum were quantified using ELISA (3). **(C)** PBMCs from five healthy donors were cultured using 50 ng/ml IL-21 and 2.5 µg/ml phosphorothioate CpG-ODN 2006 and in the presence or absence of the IgM-B-cell receptor (BCR) *in vitro* for 1 week, and EBV-specific IgG and IgM Ab levels in supernatants were quantified using ELISA. For IgM-BCR cross-linking, we cultured PBMCs (5 × 10^5^ cells/well, 24-well flat-bottomed plate) with IL-21 and phosphorothioate CpG-ODN 2006 supplemented with Affini Pure F(ab)^2^ Fragment Goat antihuman IgM (5.2 µg/ml) *in vitro* for 1 week, and IgG and IgM levels in supernatant were quantified using ELISA. The data represent five independent experiments, and graphs indicate the means ± SEMs. Significant changes from baseline were evaluated using an analysis of variance and are indicated with asterisks (*p < 0.05, **p < 0.01, ***p < 0.001).

Peripheral blood mononuclear cells were cultured as described above in the presence or absence of 2.5 µg/ml PHA-L and 15 ng/ml PMA. A comparison of IgG/IgM levels in the culture supernatants revealed significant differences in both IgG and IgM levels in the presence and absence of PHA-L and PMA (1,367.8 ± 443.1 vs. 314.6 ± 198.6 ng/ml, *p* < 0.01 and 4,540.5 ± 1,953.1 vs. 1,537.9 ± 879.4 ng/ml, *p* < 0.05, respectively) (Figure 2A).

### Evaluation of IgG/IgM EBV-Specific Abs in Culture Supernatant Using an Established *In Vitro* Assay

Both IgG and IgM EBV-specific Abs were detectable in the cultures, and significant differences in the levels of these isotypes were observed (74.99 ± 30.33 vs. 228.11 ± 70.80; *p* < 0.001). Significant differences in the serum levels of IgG and IgM isotypes were also observed (222.48 ± 154.16 vs. 70.33 ± 59.54; *p* < 0.05). We further evaluated the levels of membrane-associated Abs on non-cultured PBMCs and observed a significant difference between IgG and IgM (14.16 ± 4.18 vs. 39.22 ± 16.6; *p* < 0.05) (Figure 2B).

### Cross-Linking-Induced IgM-BCR Signaling Mediates the Class-Switching of EBV-Specific IgM mBCs and IgM Naïve B-Cells

We next investigated the effect of cross-linking-induced IgM-BCR signaling on the differentiation of EBV-specific IgM mBCs in an *in vitro* PBMC culture and not in selected mBCs.

An ELISA was used to evaluate supernatants from PBMCs cultured in the presence or absence of 5.2 µg/ml Affini Pure F(ab)^2^ Fragment Goat antihuman IgM. Significant differences in the levels of EBV-specific IgG and IgM Abs were observed between BCR-stimulated and unstimulated cultures (364.89 ± 23.27 vs. 81.57 ± 12.76 ng/ml, *p* < 0.001 and 8.3 ± 5.40 vs. 271.18 ± 10.51 ng/ml, *p* < 0.001, respectively) (Figure 2C).

### The Presence of Growth Factors and Cytokines Produced by Activated T-Cells, Macrophages, and Dendritic Cells Promoted the Differentiation of IgG and IgM mBCs into PCs

**Figure 3:**
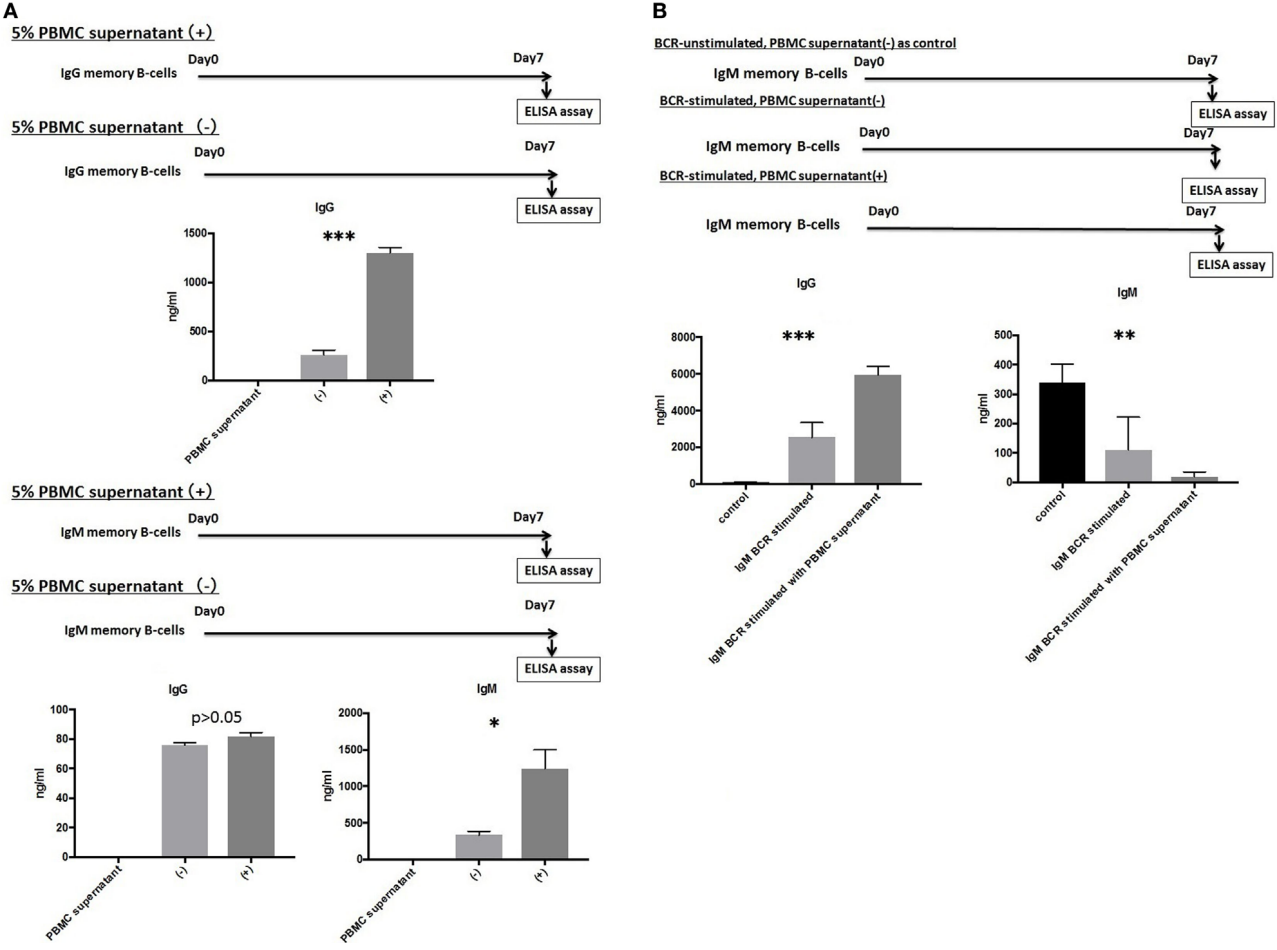
**(A)** IgG and IgM memory B-cell (mBCs) were separated from peripheral blood mononuclear cells (PBMCs) from five healthy donors and cultured with interleukin (IL)-21 and phosphorothioate CpG-oligodeoxynucleotide (ODN) 2006 and in the presence or absence of 5% PBMC supernatant *in vitro* for 1 week. IgG and IgM levels in supernatant were quantified using enzyme-linked immunosorbent assay (ELISA). **(B)** IgM mBCs separated from the PBMCs of five healthy donors were cultured *in vitro* for 1 week with IL-21, phosphorothioate CpG-ODN 2006, and one of the following conditions: IgM-B-cell receptor (BCR) stimulated with 5% PBMC supernatant; IgM-BCR stimulated without 5% PBMC supernatant; and IgM-BCR unstimulated without 5% PBMC supernatant (control). For IgM-BCR cross-linking, we cultured PBMCs (1 × 10^5^ cells/well, 24-well flat-bottomed plate) with IL-21 and phosphorothioate CpG-ODN 2006 supplemented with Affini Pure F(ab)^2^ fragment goat antihuman IgM (5.2 µg/ml) *in vitro* for 1 week. IgG and IgM levels in supernatant were quantified using ELISA. The data represent five independent experiments, and graphs indicate the means ± SEMs. Significant changes from baseline were evaluated using an analysis of variance and are indicated with asterisks (*p < 0.05, **p < 0.01, ***p < 0.001).

IgG and IgM mBCs were cultured as described above for 1 week in the presence or absence of 5% PBMCs supernatant. An ELISA analysis of culture supernatants revealed significant differences in the levels of IgG or IgM Abs produced in the presence and absence of 5% PBMC supernatant, with IgG levels (1,334.5 ± 122.0 vs.264.5 ± 89.9 ng/ml, *p* < 0.001) detected in IgG mBC culture supernatant and IgG levels (82.0 ± 5.7 vs. 76.3 ± 2.7 ng/ml, *p* > 0.05)/IgM levels (1,244.2 ± 674.0 vs. 336.3 ± 125.0 ng/ml, *p* < 0.05) detected in IgM mBC culture supernatant (Figure 3A).

### IgM-BCR Cross-Linking Is Indispensable for IgM mBC Class-Switching and Is Promoted by Growth Factors and Cytokines Produced by Activated T-Cells, Macrophages, and Dendritic Cells

We next investigated the effect of IgM-BCR cross-linking and PBMCs supernatant supplementation on the biological processes of IgM mBCs cultured *in vitro* in the presence of IL-21 and CpG-ODN. Cells were IgM-BCR stimulated or not, and cultured in the presence or absence of 5% PBMC supernatant (control: unstimulated, no PBMC supernatant). Significant intergroup differences in the levels of IgG (*p* < 0.001) and IgM (*p* < 0.01) were observed (Figure 3B).

### Detectable IgM and IgG HLA Class I and II-Specific Abs in Supernatants of Cultured PBMCs from Kidney Allograft Recipients Varied by Graft Function Status

**Figure 4:**
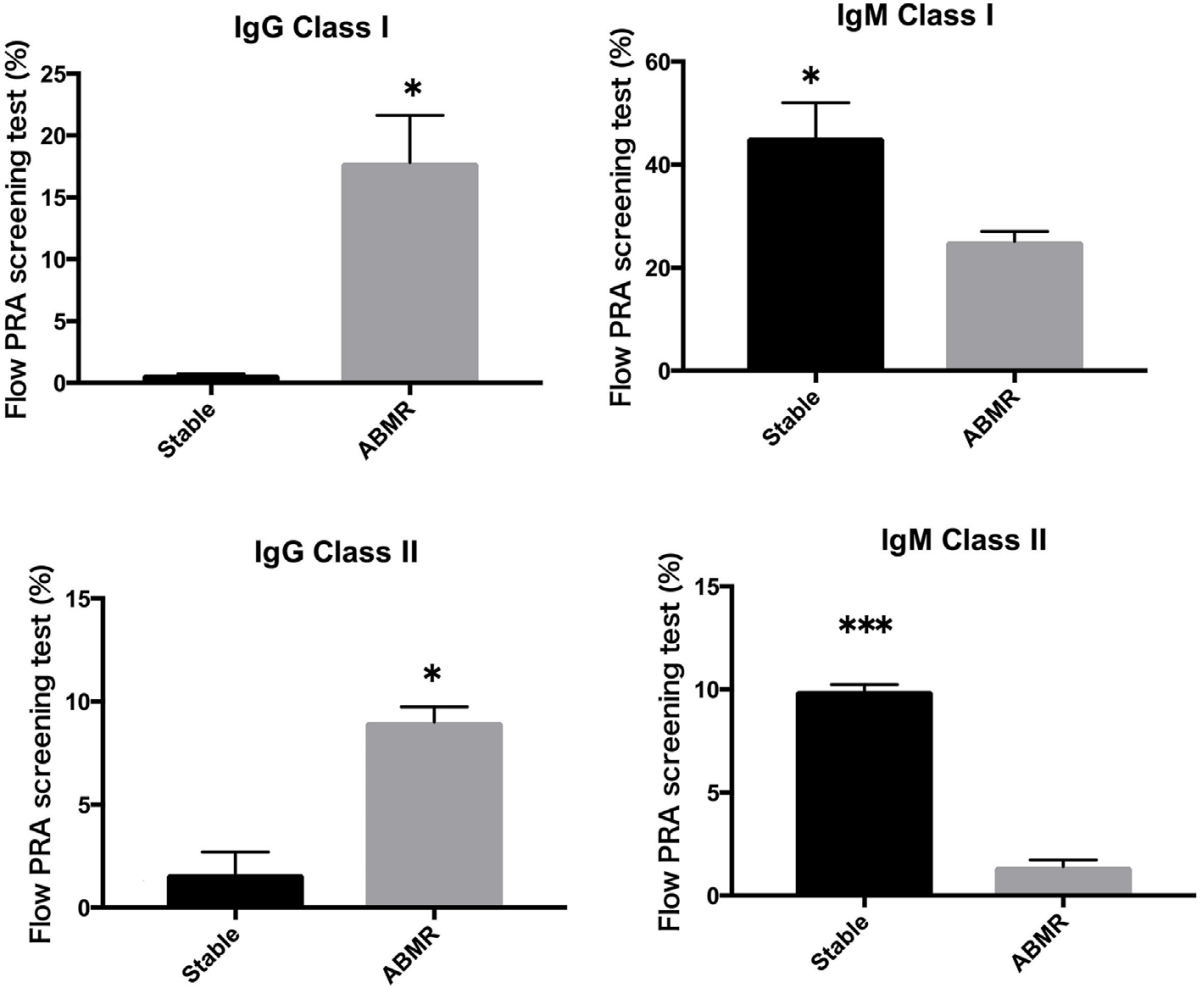
Peripheral blood mononuclear cells were collected from 16 kidney allograft recipients who were sensitized *de novo* to donor-specific antigens and cultured PBMCs from 10 healthy donors who were not sensitized to human leukocyte antigen. These were then cultured in the presence of the following additives: 50 ng/ml interleukin-21 and 2.5 µg/ml phosphorothioate CpG-oligodeoxynucleotide (ODN) 2006 in the presence of 2.5 µg/ml phytohemagglutinin/leucoagglutinin and 15 ng/ml phorbol myristate acetate *in vitro* for 1 week. The supernatants were subjected to Flow PRA screening. Stable graft function and biopsy-confirmed antibody-mediated rejection were identified using Banff scores. The data represent 16 independent experiments, and graphs indicate the means ± SEMs. Significant changes from baseline were evaluated using an analysis of variance and are indicated by asterisks (*p < 0.05, **p < 0.01, ***p < 0.001).

Peripheral blood mononuclear cells from allograft kidney recipients with stable graft function or biopsy-proven Ab-mediated rejection (ABMR) (see Table 1 for patient characteristics) and *de novo* sensitization to donor-specific antigens were cultured *in vitro* for 1 week. Subsequently, the cultured supernatants were analyzed using a Flow PRA screening test. A comparison of IgG and IgM HLA Abs levels (%) in the supernatants of these cultures revealed significant differences related to graft function stability (class I: *p* < 0.05, *p* < 0.05, respectively; class II: *p* < 0.05, *p* < 0.001, respectively; Figure 4).

**Table 1:** Patient characteristics.

Parameter		Graft function stable (*n* = 7)	Ab-mediated rejection (*n* = 9)	*p*-Value
Gender (male/female)	N(%)	2 (28.5%)/5 (71.4%)	6 (66.7%)/3 (33.3%)	N.S.
Age at transplant (year)	Mean ± SD	42.5 ± 4.0	40.2 ± 19.8	N.S.
Immunologic characteristics				
DSA (strong = 1/moderate = 2/weak = 3)	*N* (%)	2 (28.5)/2 (28.5)/3 (42.6)	5 (55.6%)/4 (44.4%)/0 (0%)	N.S.
*De novo* DSA type (class 1 = 1/class2 = 2/class1 + class2 = 3)	*N* (%)	2 (28.5%)/3 (42.6)/2 (28.5)	2 (22.2%)/4 (44.4%)/3 (33.3%)	N.S.
BANFF SCORE				
CD40 (negative = 1/positive = 2)	*N* (%)	7 (100%)/0 (0%)	0 (0%)/9 (100%)	<0.001***
g + ptc	Mean ± SD	0.4 ± 0.5	3.5 ± 1.0	<0.001***

### Higher Frequencies of DSA-Specific IgM mBCs Relative to IgG mBCs Are Usually Observed in the Peripheral Blood

**Figure 5:**
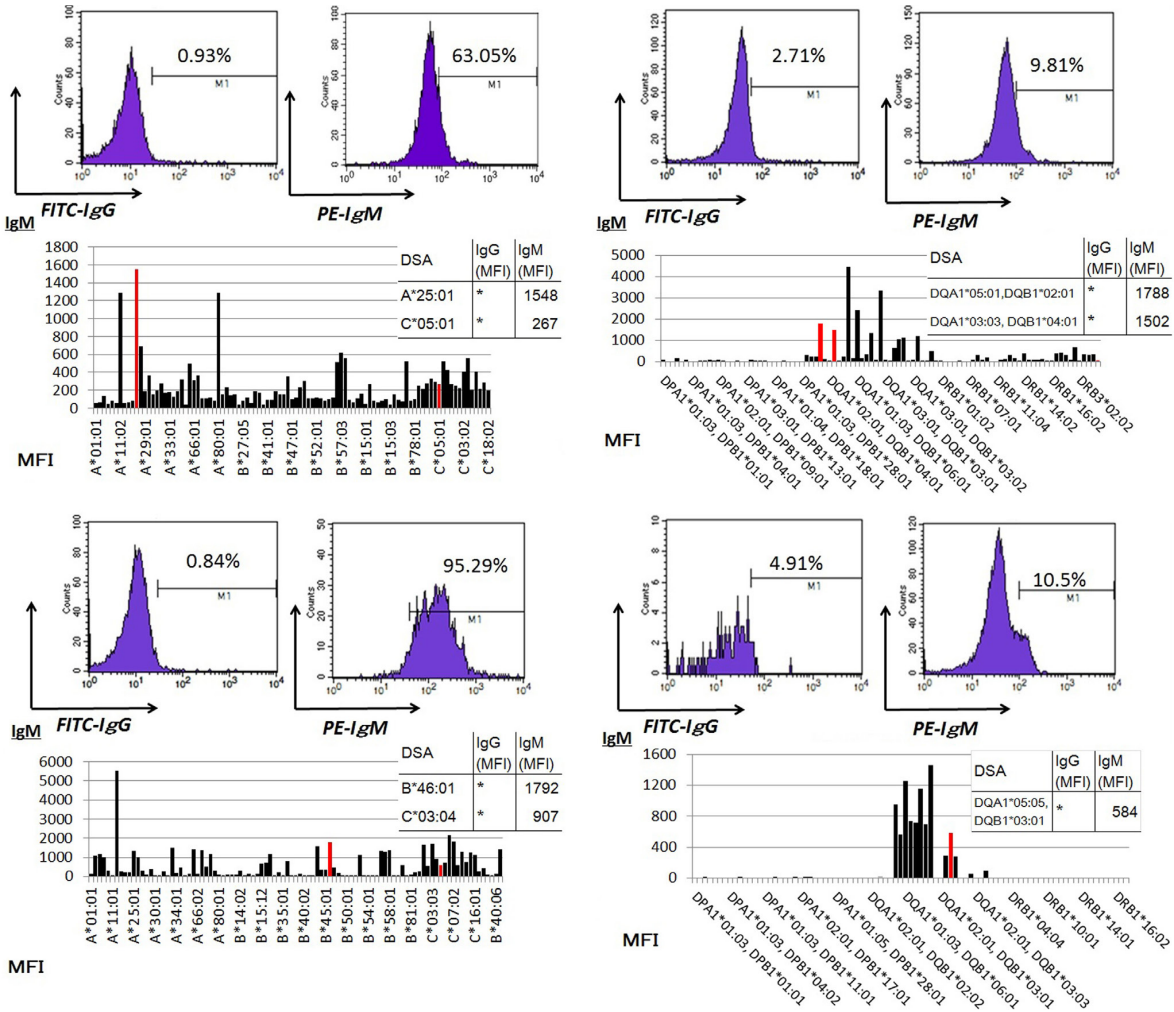
Peripheral blood mononuclear cells were cultured using 50 ng/ml interleukin-21 and 2.5 µg/ml phosphorothioate CpG-oligodeoxynucleotide 2006 in the presence of 2.5 µg/ml phytohemagglutinin/leucoagglutinin and 15 ng/ml phorbol myristate acetate *in vitro* for 1 week. The supernatants were subjected to Flow PRA screening, and positive cases underwent an additional Luminex single-antigen beads evaluation. PBMCs were collected from four kidney allograft recipients with stable graft function who were sensitized *de novo* to donor-specific antigens and cultured under *in vitro* assay conditions. The data represent four independent experiments. The red column indicates DSAs. * indicates that human leukocyte antigen antibodies were not detected by Flow PRA screening and the supernatant was not analyzed by Luminex single-antigen bead evaluation.

Peripheral blood mononuclear cells from allograft kidney recipients with *de novo* donor-specific antigen sensitization and stable graft function were cultured *in vitro* for 1 week. A Luminex single-antigen bead analysis mainly detected IgM DSAs the culture supernatants (Figure 5).

### IgG DSA Production in PBMC Culture Increases following the *In Vivo* Activation of Humoral Immunity against a Donor-Specific Antigen

**Figure 6:**
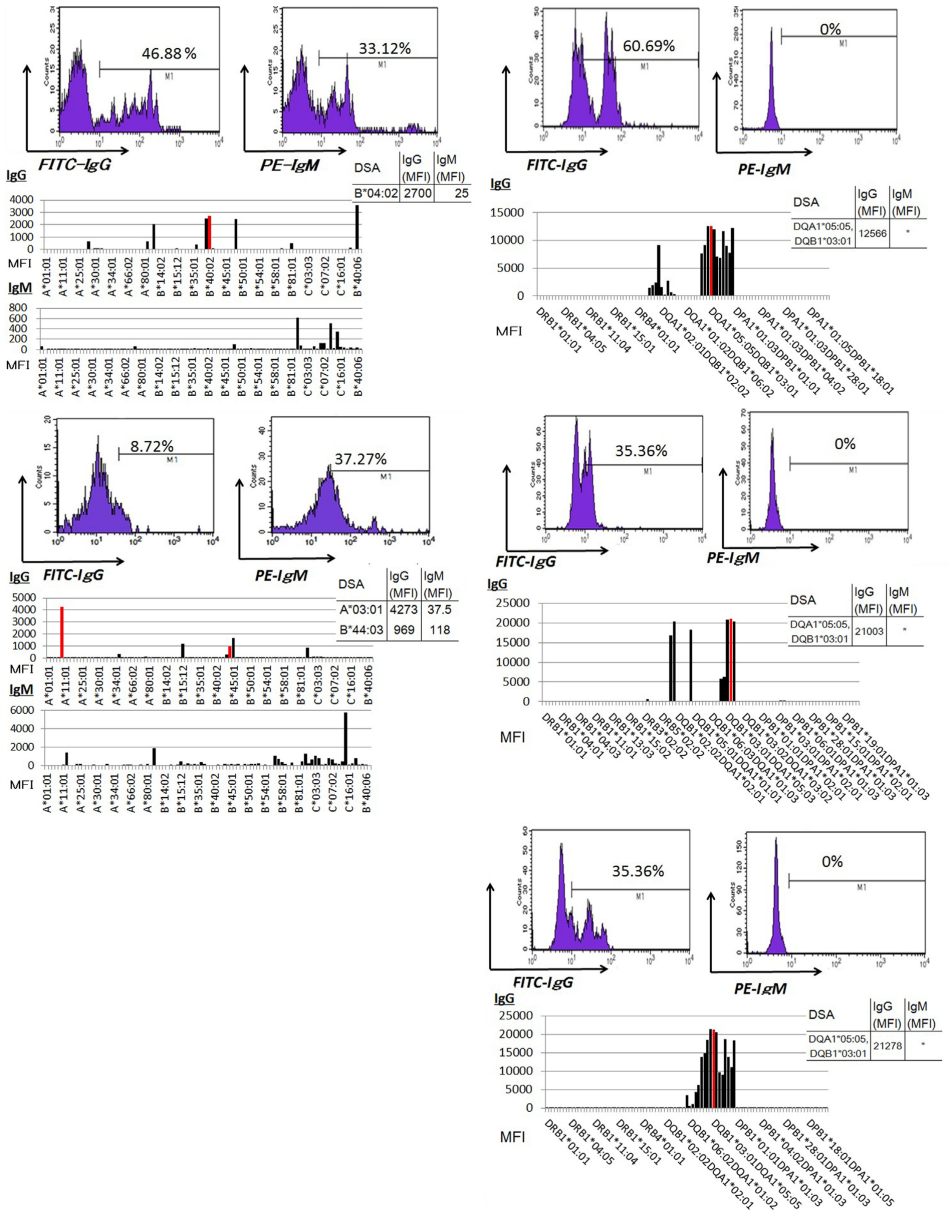
Peripheral blood mononuclear cells were cultured using 50 ng/ml interleukin-21 and 2.5 µg/ml phosphorothioate CpG-oligodeoxynucleotide 2006 in the presence of 2.5 µg/ml phytohemagglutinin/leucoagglutinin and 15 ng/ml phorbol myristate acetate *in vitro* for 1 week. The supernatants were subjected to Flow PRA screening, and positive cases underwent an additional Luminex single-antigen beads evaluation. PBMCs from five kidney allograft recipients with biopsy-confirmed antibody-mediated rejection were cultured under *in vitro* assay. The data represent five independent experiments. The red column indicates DSAs. * indicates that human leukocyte antigen antibodies were not detected by Flow PRA screening and the supernatant was not analyzed by Luminex single-antigen bead evaluation.

Peripheral blood mononuclear cells from allograft kidney recipients with *de novo* donor-specific antigen sensitization and biopsy-confirmed ABMR were cultured *in vitro* for 1 week. A Luminex analysis of culture supernatants mainly detected IgG DSAs (Figure 6).

### IgG-Type DSAs Were Detectable in the Serum Regardless of Humoral Immune Activity against Donor-Specific Antigens, Whereas IgM Type DSA Detection Was Unreliable

**Figure 7:**
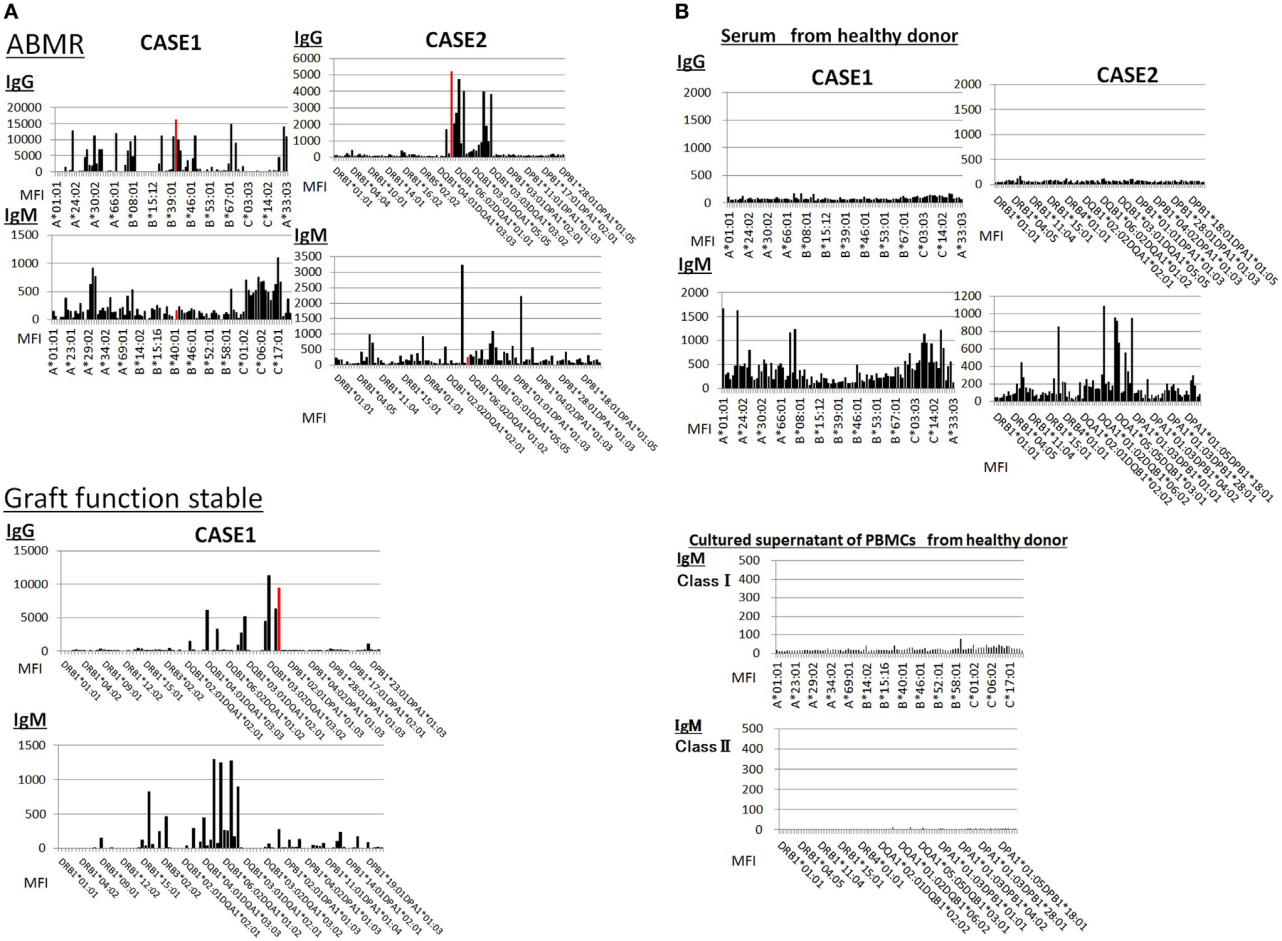
**(A)** Serum samples were collected from one kidney allograft recipients with stable graft function and two kidney allograft recipients with biopsy-proven Ab-mediated rejection (ABMR) who were sensitized *de novo* to donor-specific antigens and subjected to a Luminex single-antigen evaluation. The data represent six independent experiments. The red column indicates DSAs. **(B)** Serum samples were collected from two healthy donors who were not sensitized to human leukocyte antigens and subjected to a Luminex single-antigen evaluation. Peripheral blood mononuclear cells (PBMCs) obtained from healthy donors who were not sensitized by human leukocyte antigen were cultured using 50 ng/ml interleukin -21 and 2.5 µg/ml phosphorothioate CpG-oligodeoxynucleotide 2006 in the presence of 2.5 µg/ml phytohemagglutinin/leucoagglutinin and 15 ng/ml phorbol myristate acetate *in vitro* for 1 week, and culture supernatants were analyzed by Luminex single-antigen evaluation. The data represent six independent experiments.

Sera from donor-specific antigen-sensitized and non-sensitized healthy donors were subjected to a Luminex analysis. IgG DSAs were detectable in sera from one allograft kidney recipient with stable graft function and two allograft kidney recipients with biopsy-proven antibody-mediated rejection (Figure 7A). By contrast, the level of IgM type DSAs was significantly lower compared to that of IgG- and many IgM type HLA Abs were detected broadly in the sera. In addition, IgM HLAs were detected in the sera from non-sensitized healthy donors; these were not detected in PBMC culture supernatants (Figure 7B).

## Discussion

In context of transplantation, the development of an *in vitro* T-cell assay for drug sensitivity has facilitated better control of T-cell-mediated rejection, which occurs during the early post-procedural stage (16, 17). By contrast, antibody-mediated rejection has not been well addressed, and effective early diagnostic and treatment methods have not been developed (18–21). Currently, no available humoral immunosuppressive agent can control PC growth and survival, as these cells can survive independently of helper T-cells (22). Furthermore, Abs produced by these PCs might be absorbed into target tissues or form complexes with target antigens (23, 24), thus causing irreversible disease progression (25, 26).

Therefore, a method to detect mBCs prior to PC differentiation is urgently needed to ensure the timely initiation of essential treatment. Fortunately, the identification and clarification of the target antigens of Ab-associated diseases should theoretically allow the detection of specific Ab-producing mBCs in the periphery using minimally invasive diagnostic methods.

Some reports suggest that enzyme-linked immunospot assays can be used to detect antigen-specific mBCs (27, 28). However, we further expect that a useful *in vitro* assay for the analysis of mBCs-derived Abs in the supernatants will allow drug sensitivity tests, evaluate the differentiation of mBCs into PCs, and contribute to the development of more effective treatments for controlling Ab-associated disease.

Previously, Haneda et al. successfully demonstrated the evaluation of immunosuppressive sensitivity in an *in vitro* B-cell assay originally developed by Jourdan et al. (29) to quantitate IgG in culture supernatants (30). We note, however, that the total B-cell population includes both naïve B-cells and mBCs (31, 32). Because IL-21, CD40 ligand, and CpG-ODN have been reported to induce IgG class-switching *in vitro*, IgG Abs from a total B-cell population might also include naïve B-cell-derived IgG Abs (33–37). We, therefore, urge the use of selective assays to evaluate specifically mBCs-derived Abs in culture supernatants.

Some researchers have attempted to induce the *in vitro* differentiation of CD19^+^ B-cells into PCs during analyses of targeted antigen-specific Abs in culture supernatants. However, we found many reports stating that mBCs require assistance from antigen-specific helper T-cells in a cell–cell contact-dependent manner (38–42). Therefore, we attempted to more efficiently culture PBMCs *in vitro* to collect mBC-derived Abs from culture supernatants.

In our PBMC cultures, PHA-L was used to activate helper T-cells, which subsequently produced growth factors and cytokines (including CD40 ligand). PMA, a tyrosine-protein kinase activator, was added to promote TNF-α production from macrophages (43). CpG-ODN was added to induce B-cell proliferation and promote dendritic cell and macrophage maturation by binding to TLR-7, ultimately leading to helper T-cell activation. IL-21 was added to promote the differentiation of mBCs into PCs after stimulation through the BCR and CD40; this cytokine also activates helper T-cells through IL-21 receptor (44, 45). In summary, we expect that our *in vitro* culture conditions will be more suitable for mBC growth and survival, compared to previously reported conditions.

We further note that mBC-derived Abs should be collected selectively to address the possibility that natural Abs, including naïve B-cell-derived IgM Abs might inhibit the detection of targeted antigen-specific Abs in form of pentamers (46, 47). Despite a lack of previous stimulation, naïve B-cells possess the ability to produce IgM Abs against foreign antigens within context of a primary immune response. We, therefore, focused on differences in the periods required for the differentiation of naïve B-cells and mBCs into PCs. As mBCs have already been sensitized to antigen, they would be expected to differentiate more rapidly (48, 49). Our *in vitro* experiments demonstrated that mBCs were stimulated to differentiate into PCs by day 7, or earlier than naïve B-cells.

We further note that our cultures might have contained the TNF ligand superfamily member, APRIL, which is produced by activated macrophages and dendritic cells (50). According to many reports, APRIL supports B-cell proliferation and differentiation, especially during later stage differentiation (51–56). Consequently, the proportions of naïve B-cell-derived Abs in our supernatants may have increased as the culture period increased. Therefore, we focused on the IgG and IgM levels in supernatants from culture day 7 as the most suitable parameter and time point for mBC-derived Abs evaluations after confirming that stimulated CD27^+^ mBCs were found to differentiate into PCs significantly earlier, compared with CD27^−^ naïve B-cells at that time (Figure 1A).

In this study, we further confirmed our ability to detect both IgG and IgM EBV-specific Abs in culture supernatants using our established *in vitro* assay, an important point given the high rate of EBV infection history in the population (>90% of healthy adult donors). For this analysis, we took care to enroll only healthy donors who did not exhibit signs of active EBV infection (e.g., fever, swollen lymph nodes, and hepatitis) (14, 57, 58). According to our results, a stable humoral immune reaction against EBV correlated with a higher ratio of peripheral blood IgM mBCs relative to IgG mBCs, suggesting that antigen-specific IgM might be more easily detectable when using a smaller volume of blood (8 ml). By contrast, some reports have indicated that a large quantity of peripheral blood (>20 ml) (12, 14, 26) might be needed to reliably detect antigen-specific IgG in culture supernatants because the frequency of antigen-specific IgG mBCs might vary greatly according to the activation status of an antigen-specific humoral immune response (27).

In addition, our *in vitro* IgM mBC assay demonstrated that cross-linking-induced IgM-BCR stimulation is indispensable for the class-switching of IgM to IgG, and that growth factors and cytokines produced by activated T-cells, macrophages, and dendritic cells promoted this process. These results suggest that circulating IgM mBCs can undergo class-switching to become IgG mBCs and could thereby cause tissue injury following an antigen–BCR reaction, as BCR cross-linking-mediated stimulation increases the generation of antigen-specific IgG mBCs in the peripheral blood (Figure S1 in Supplementary Material).

For *in vivo* analysis, we used PBMCs from allograft kidney recipients sensitized *de novo* to donor-specific antigens because the activation of a humoral immune reaction against these antigens can be evaluated accurately through a kidney biopsy. We found that IgG DSAs were only detectable in the culture supernatants from the cells of kidney allograft recipients with biopsy-proven ABMR, whereas IgM Abs were generally detectable in supernatants from the cells of patients with stable graft function.

On the contrary, PCs in the bone marrow maintain long-term production of antigen-specific IgG Abs in the serum regardless of the antigen–antibody reaction status.

Regarding IgM Abs, we examined the effect of natural IgM Abs on the detection of antigen-specific IgM and found that some IgM HLA Abs were detectable in the sera from healthy donors who had not been sensitized to HLA (59, 60). These false positives indicated the possibility that the patients’ natural Abs could have reacted non-specifically with the Luminex single-antigen beads (Figure 7B).

In addition, the level of IgM-type DSAs was significantly lower than that of IgG type, and many IgM-type HLA Abs were broadly detected in the sera from kidney recipients sensitized with *de novo* DSAs; it was very difficult to determine whether DSA was produced in serum. Thus, it might indicate that natural IgM Abs might inhibit the detection of IgM DSAs or specific *in vivo* conditions might be necessary for DSA-specific IgM mBCs differentiation into PCs.

By contrast, we could not detect IgM HLA Abs in the culture supernatants of PBMCs from these healthy donors (Figure 7B). Therefore, our culture condition might not be ideal for the production of natural Abs, whereas IgM (IgD^+^) mBCs differentiate selectively into PCs through a GC -dependent process and produce antigen-specific Abs.

In conclusion, the simultaneous detection of antigen-specific IgG and IgM from PBMC culture supernatant may provide a more effective and accurate minimally invasive evaluation of Ab-associated pathological conditions, compared to the detection of each type of antigen-specific Ab from the serum.

Furthermore, the establishment of an *in vitro* assay to collect both antigen-specific IgG and IgM Abs has a possibility of allowing immunosuppressive agent susceptibility tests targeting the differentiation of each type of mBC into PCs. Thus, the combination of detected Abs in the culture supernatant may contribute in introducing a more individualized immunosuppressive therapy.

Additionally, we confirmed the class-switching of IgM mBCs *in vitro*, and showed that immunosuppressive agent susceptibility tests targeting class-switching from IgM mBCs to IgG are also possible.

In other words, there is a possibility that this study may also be applied to the development of more effective immunosuppressive therapies corresponding to the level of progression of the disease state in context of Ab-associated diseases.

## Ethics Statement

This study was carried out in accordance with the ethics committee of Osaka University. All subjects gave written informed consent in accordance with the Declaration of Helsinki. The protocol was approved by the ethics committee of Osaka University.

## Author Contributions

YM designed and wrote paper, did all experiments. RI and ST provided excellent advice.

## Conflict of Interest Statement

The authors declare that the research was conducted in the absence of any commercial of financial relationship that could be construed as a potential conflict of interest.

## Supplementary Material

Figure S1 Peripheral blood mononuclear cell (PBMCs) collected from two kidney allograft recipients sensitized to *de novo* DSAs were cultured with interleukin-21 and phosphorothioate CpG-oligodeoxynucleotide 2006 and in the presence or absence of Affini Pure F(ab)^2^ fragment goat antihuman IgM (5.2 µg/ml) *in vitro* for 1 week. IgM cross-linking was performed on PBMC culture and not on selected memory B-cells. Culture supernatants were subjected to Flow PRA and Luminex single-antigen beads. The mean fluorescence intensity (MFI) of each human leukocyte antigen antibody from the PBMC-cultured supernatant was compared between B-cell receptor (BCR)-stimulated and -unstimulated culture conditions, and the MFI of the latter was divided by that of the former. The ratios are shown in the graph. Data represent two independent experiments. Red column shows DSAs.
