# Supplementary figures and images for: Evaluation of Antigen-Specific IgM and IgG Production during an In Vitro Peripheral Blood Mononuclear Cell Culture Assay
Source: Front Immunol. 2017 Jul 10;8:794. doi: 10.3389/fimmu.2017.00794 (PMC5502262; doi:10.3389/fimmu.2017.00794)

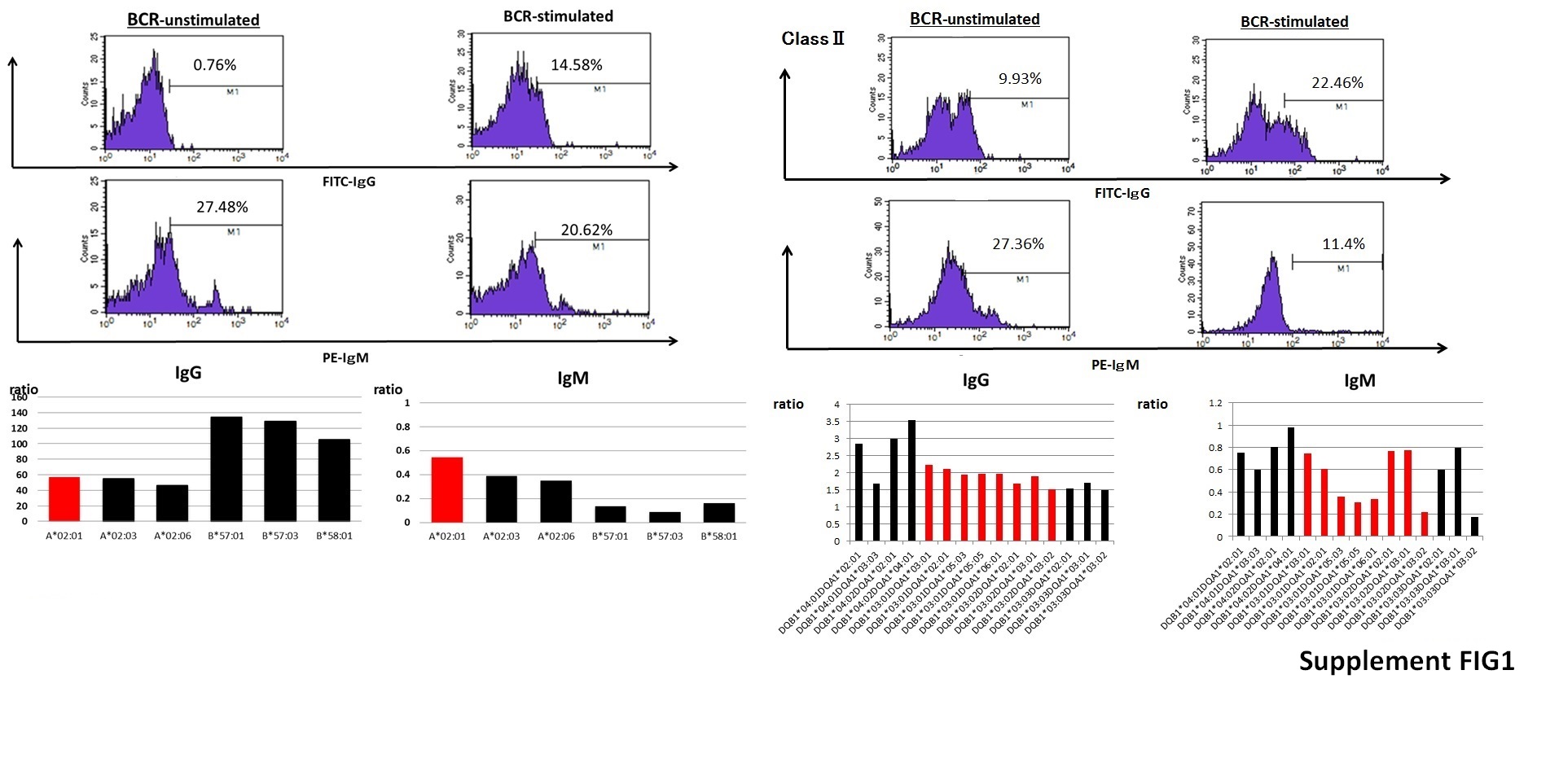

Supplement: Figure S1 — Peripheral blood mononuclear cell (PBMCs) collected from two kidney allograft recipients sensitized to de novo DSAs were cultured with interleukin-21 and phosphorothioate CpG-oligodeoxynucleotide 2006 and in the presence or absence of Affini Pure F(ab)2 fragment goat antihuman IgM (5.2 µg/ml) in vitro for 1 week. IgM cross-linking was performed on PBMC culture and not on selected memory B-cells. Culture supernatants were subjected to Flow PRA and Luminex single-antigen beads. The mean fluorescence intensity (MFI) of each human leukocyte antigen antibody from the PBMC-cultured supernatant was compared between B-cell receptor (BCR)-stimulated and -unstimulated culture conditions, and the MFI of the latter was divided by that of the former. The ratios are shown in the graph. Data represent two independent experiments. Red column shows DSAs. [file Image_1.JPEG]
